# Harmful algal bloom-related 311 calls, Cape Coral, Florida 2018–2019

**DOI:** 10.2166/wh.2022.257

**Published:** 2022-03

**Authors:** Christopher K. Uejio, Elaina Gonsoroski, Samendra P. Sherchan, Leslie Beitsch, E. W. Harville, C. Blackmore, K. Pan, Maureen Y. Lichtveld

**Affiliations:** a Department of Geography, College of Social Sciences and Public Policy, Florida State University, 113 Collegiate Loop, Tallahassee, FL 32306, USA; b Department of Environmental Health Sciences, School of Public Health and Tropical Medicine, Tulane University, 1440 Canal Street, Suite 2100, New Orleans, LA 70112, USA; c Department of Behavioral Sciences and Social Medicine, College of Medicine, Florida State University, 115 W Call St, Tallahassee, FL 32304, USA; d Department of Epidemiology, School of Public Health and Tropical Medicine, Tulane University, 1440 Canal Street, Suite 2000, New Orleans, LA 70112, USA; e Division of Disease Control and Health Protection, Florida Department of Health, 4052 Bald Cypress Way, Tallahassee, FL 32399, USA; f Department of Environmental and Occupational Health, Graduate School of Public Health, University of Pittsburgh, 130 DeSoto Street, Pittsburgh, PA 15261, USA

**Keywords:** Florida, human health risk, policy, water quality

## Abstract

Harmful algal blooms (HABs) can adversely impact water quality and threaten human and animal health. People working or living along waterways with prolonged HAB contamination may face elevated toxin exposures and breathing complications. Monitoring HABs and potential adverse human health effects is notoriously difficult due to routes and levels of exposure that vary widely across time and space. This study examines the utility of 311 calls to enhance HAB surveillance and monitoring. The study focuses on Cape Coral, FL, USA, located along the banks of the Caloosahatchee River and Estuary and the Gulf of Mexico. The wider study area experienced a prolonged cyanobacteria bloom in 2018. The present study examines the relationship between weekly water quality characteristics (temperature, dissolved oxygen, pH, microcystin-LR) and municipal requests for information or services (algal 311 calls). Each 1 μg/L increase in waterborne microcystin-LR concentrations corresponded with 9% more algal 311 calls (95% confidence interval: 1.03–1.15, *p* = 0.002). The results suggest water quality monitoring and the 311 dispatch systems may be further integrated to improve public health surveillance.

## INTRODUCTION

Harmful algal blooms (HABs) can adversely impact drinking and recreational water quality and threaten human and animal health (Cheung *et al*. 2013). HABs also have broader negative economic impacts on livestock, fishing and aquaculture, recreation and tourism, property values, drinking water treatment and monitoring, and ecosystem services (Hamilton *et al*. 2014). Globally, the frequency, duration, and geographic range of HABs are increasing (Glibert & Burford 2017).

Freshwater and estuarine cyanobacteria have multiple qualities that promote algal blooms. Cyanobacteria have cellular and colony buoyancy strategies that allow rapid colony formation on the water’s surface (Wagner & Adrian 2009). Excess phosphorus pollution can initiate phytoplankton blooms which create conditions (decreased light, increased carbon dioxide) that indirectly promote cyanobacteria (Hyenstrand *et al*. 1998; Downing *et al*. 2001). Cyanobacteria’s ecophysiological adaptations complexly interact with hydrologic conditions. In shallow Florida (USA) lakes, multi-year dry (wet) periods promoted (suppressed) blooms, while individual rainfall events also decreased blooms (Havens *et al*. 2019).

The most common human cyanobacteria exposure pathways are consuming contaminated water/food or dermal contact during recreational activities. These exposures can produce acute gastrointestinal, neurologic, renal, or dermal symptoms (e.g. Backer *et al*. 2015). There is also emerging evidence that blue-green algae, microorganisms, and cyanotoxins may become airborne (aerosolized) and cause respiratory distress (Stewart *et al*. 2006a, 2006b; Backer *et al*. 2010; Lewandowska *et al*. 2017; May *et al*. 2018). Microcystin-LR is one of the most toxic and common types of cyanotoxin emitted by cyanobacteria. People working or living along waterways may face elevated exposures but cyanobacteria exposures are also common further inland (Facciponte *et al*. 2018).

Simultaneously monitoring dynamic water and airborne cyanotoxin exposures and subclinical adverse human health outcomes requires substantial resources and serendipity. The present study investigates how cyanobacterial bloom water quality monitoring and the 311 dispatch systems may be further integrated to improve public health surveillance. As part of a larger ‘smart cities’ movement, municipalities created 311 call centers to streamline residents’ requests for government services and increase transparency (e.g. Harrison & Donnelly 2011; Reddick 2011, Nam & Pardo 2014). In spite of their clear connections to societal determinants of health, researchers are just starting to explore how analyzing 311 calls may improve human health and well-being (e.g. Athens *et al*. 2020).

The study investigates the extent to which 311 calls can help identify potential adverse health effects of cyanobacterial blooms that may not be apparent from existing health surveillance systems (e.g. syndromic surveillance; One Health Harmful Algal Bloom System) due to low-dose exposures and subtle health effects. Specifically, the study examines the relationship between water quality characteristics (e.g. temperature, dissolved oxygen, pH, microcystin-LR) and 311 cyanobacterial bloom calls in Cape Coral, FL, USA from June 2018 to October 2019. This analysis presumes that the Cape Coral algal 311 calls are primarily related to the freshwater cyanobacteria rather than the marine *Karenia brevis* bloom in the nearby Gulf of Mexico (Metcalf *et al*. 2021).

## METHODS

### Study site

**Figure 1 |:**
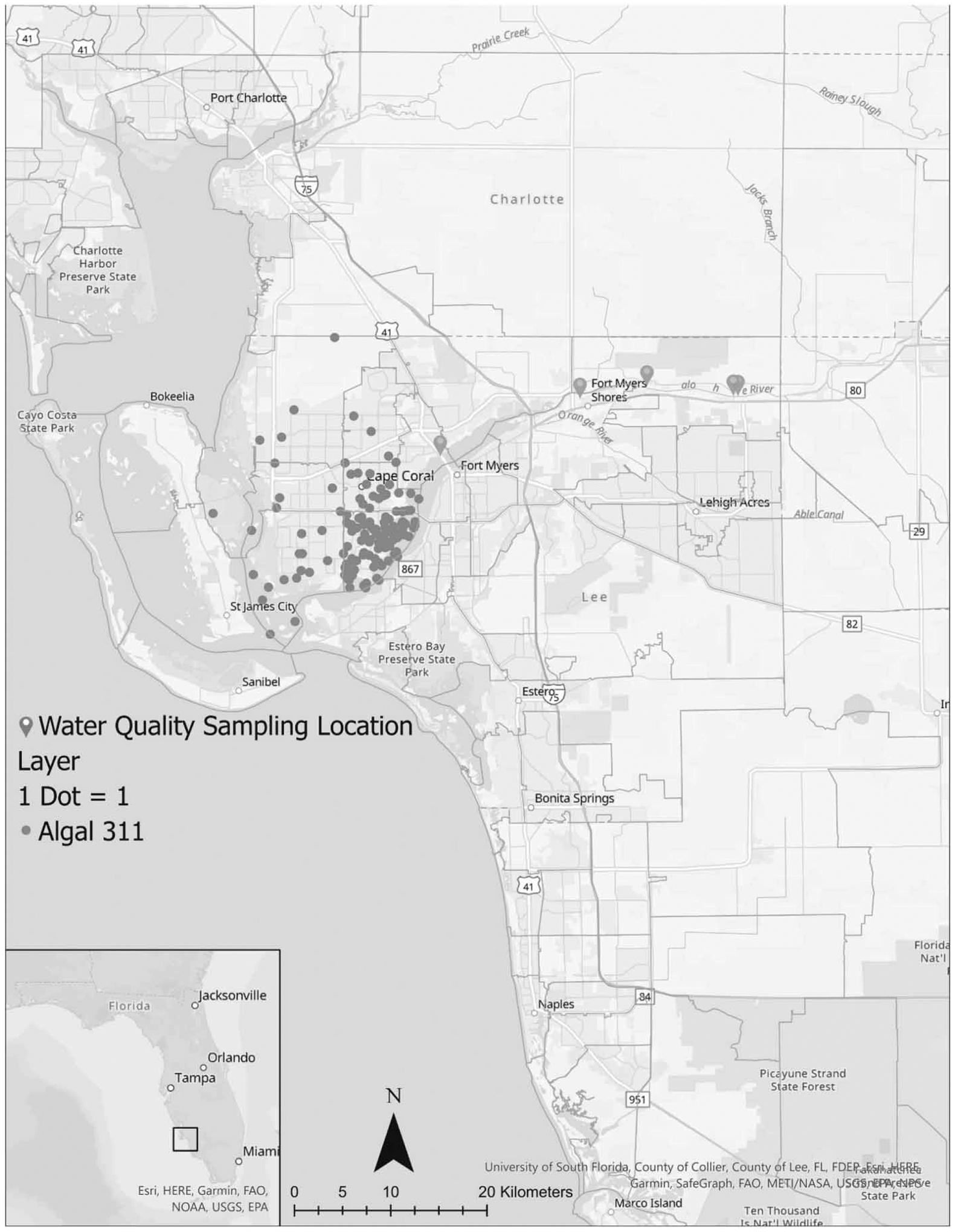
Dot density map illustrating the distribution of algal 311 calls by postal zip codes and upstream water quality sampling locations. The 311 locations have been randomized within their respective zip codes to protect privacy. The city of Cape Coral is sandwiched between the Caloosahatchee River and Estuary, FL, and the Gulf of Mexico.

The City of Cape Coral was located in southwest Florida, USA, between the Caloosahatchee River and Estuary and the Gulf of Mexico (Figure 1). The river’s hydrology was substantially altered by dredging, channelization, water extraction, and new connections to Lakes Hicpochee and Okeechobee (Antonini *et al*. 2002). The river’s width ranged from 50 to 130 m, while the typical depth was between 6 and 9 m (Barnes 2005). Freshwater cyanobacteria blooms were more likely to occur during rainy periods that increase phosphorus and nitrogen runoff and limit tidal flushing (Krimsky *et al*. 2018).

On occasion, Lake Okeechobee’s water were discharged into the Gulf of Mexico through the Caloosahatchee River and to the Atlantic Ocean through the St. Lucie River (Canfield *et al*. 2021). In 2018, Lake Okeechobee, a eutrophic lake with excess plant and algae growth and nutrient availability, discharged 1.3 million acre-feet into the Caloosahatchee River and 0.6 million acre-feet into the St. Lucie River (South Florida Water Management District 2019). These discharge events promoted blooms on the St. Lucie River but were less studied in the Caloosahatchee River (Phlips *et al*. 2012).

### Data sources

#### 311 calls

The City of Cape Coral provided 311 call information for 2018–2019 (City of Cape Coral 2020). The investigators defined 311 algal calls as those with keywords in the call title or call description fields. While some of the keywords are more specifically related to cyanobacteria (Blue Agal, AGAL BL, BGA, reportalgalbloom.com), most records (72%) non-specifically discuss harmful algae (algae, Algae, ALGAE, algal, ALGAL, Algal). Thus, this study could not distinguish between cyanobacteria and other HABs. The call center directed callers to the Florida Department of Environmental Protection’s (FDEP) website (reportalgalbloom.com), which contains a one-question survey for reporting blooms. Of the remaining 311 calls, the study excluded calls containing ‘Swimming Pool’ or ‘pool’ keywords, which likely referred to unmaintained pools instead of river/canal water quality. The Cape Coral 311 system accepts calls from both residents and non-residents. Most (66%) of the submissions voluntarily reported a Cape Coral incident address, while the remaining callers did not report this information.

#### Water quality samples

The FDEP shared cyanobacteria and water quality sampling data from 2018 to 2019 (Florida Department of Environmental Protection 2020). The state laboratory measured the water sample’s microcystin-LR concentrations (μg/L) and dominant cyanobacteria taxa. The FDEP also consistently measured dissolved oxygen (mg/L), specific conductance (umhos/cm), temperature (°C), and pH. The study investigator’s quality control procedures removed flagged data values below the detection limit/quantification value, improperly or unpreserved samples, estimated values, and potentially contaminated samples.

The FDEP prioritizes cyanobacteria sampling based on potential human exposure, converging public reports, and site sampling history. To meet the multiple surveillance goals, the FDEP repeatedly sampled water quality from five upstream locations (Figure 1; Alva Boat Ramp, Davis Boat Ramp, Franklin Locks, S79, North Shore Park). The microcystin-LR concentrations were generally related (median Spearman’s Rho: 0.59, range: 0.24–0.74) between the five longitudinally water quality sites spread out over 10 km. Thus, the study created weekly average water quality from these locations. The dataset was further restricted to weeks with sequential water quality samples (sampled in the preceding week) to control for temporal autocorrelation. In the dataset, water quality was measured over a greater proportion of the year in 2019 (69.2% of weeks, week of the year range: 2–51) than in 2018 (36.5% of weeks, week of the year range: 25–51).

#### Analysis

Summary statistics reported the weekly 311 algal call’s mean, variance, and range and average weekly water quality sample’s microcystin-LR concentrations, dissolved oxygen levels, specific conductance, temperature, and pH. The analysis was conducted in the R statistical computing and analysis program (2020, version 4.0.2). A word cloud plotted the most commonly used 311 call description field words, where the size of the word was scaled to the frequency of occurrence. Data preprocessing converted removed numbers, sequential white space characters, punctuation, and stop words – commonly used English language pronouns and prepositions (ISO 639–1).

Negative binomial regression models (MASS package) regressed average weekly water quality (dissolved oxygen, specific conductance, temperature, pH, and microcystin-LR) against the count of algal 311 calls over 2018–2019 (Venables & Ripley 2002). To control for unexplained temporal autocorrelation, the statistical model included the previous week’s 311 call count (natural logarithm transformed +1) and an indicator variable for the study year (2019 versus 2018). Auto and partial-autocorrelation plots of the model residuals verified that residual autocorrelation was adequately controlled. The study reports the results as odds ratios, standard errors, *p*-values, and 95% confidence intervals. The odds ratio quantifies the strength of the association between water quality conditions, temporal characteristics, and algal 311 calls. An odds ratio of 1 implies no relationship between independent variables and 311 calls. Correspondingly, odds ratios notably greater than 1 (less than 1) suggest water quality/temporal characteristics are positively (negatively) associated with 311 calls. This analysis presumes: (a) local residents made the majority of 311 calls, (b) the HAB calls were primarily related to cyanobacteria, (c) upstream (10 km) water quality was representative of local risks, and (d) water and airborne microcystin-LR concentrations were related. The analysis dataset is archived at DOI:10.5281/zenodo.4966946.

## RESULTS

**Figure 2 |:**
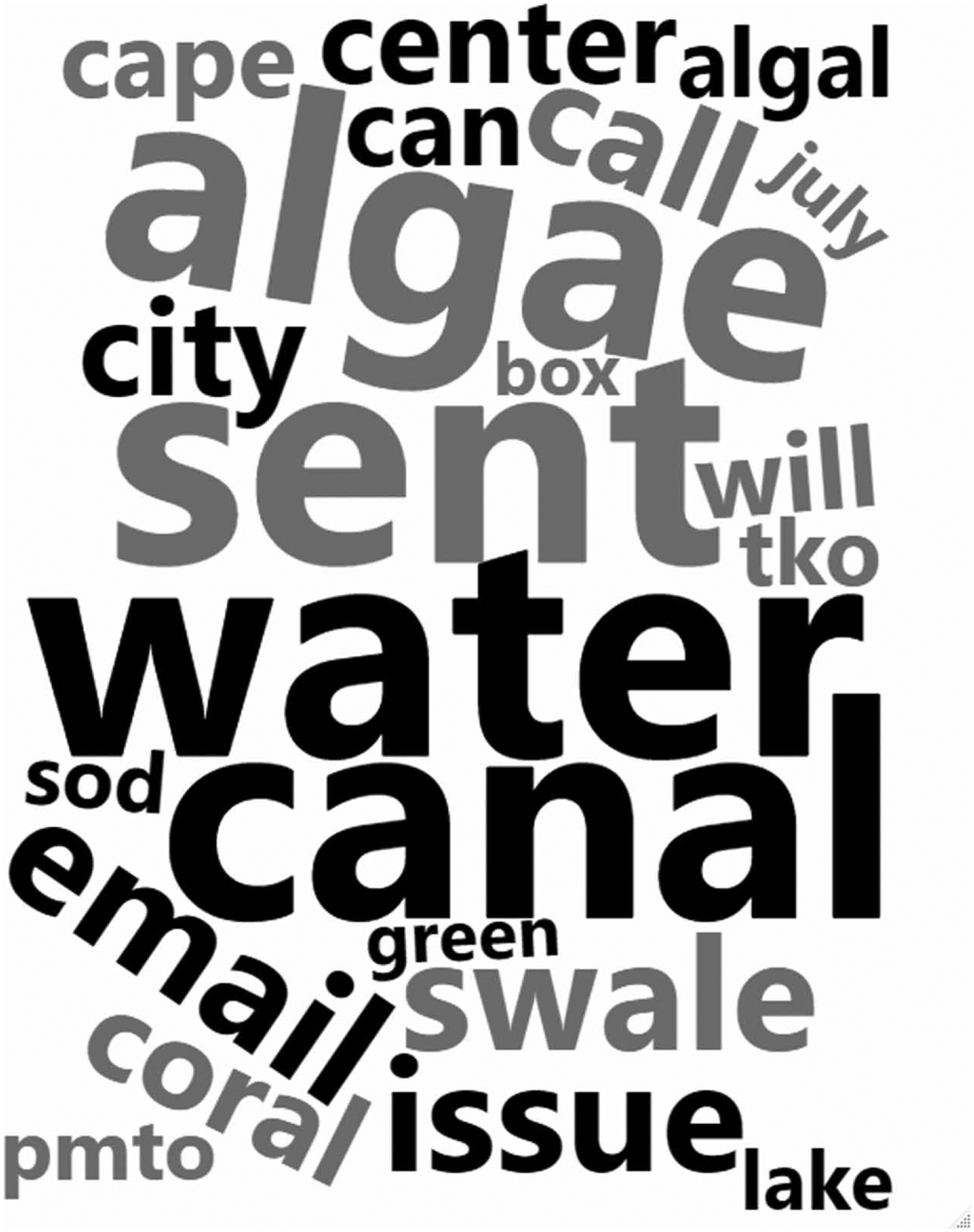
A cloud of words used 37 or more times in the 311 free text call description field. The word cloud highlights water quality (algae, algal, bloom), infrastructure (canal, swale), and city service reply keywords (please, email, call).

Most of the year, Cape Coral only received one algal 311 call each week. However, call volume exhibited clear seasonality with a peak from July to September each year. The largest number of calls (41) was recorded during the week of August 5, 2018. Thus, 311 calls followed an over dispersed (variance notably greater than the mean) count distribution (mean = 3.5, variance = 49.1, total calls = 194). A word cloud plots words referenced 37 or more times in the free text description field (Figure 2). The plot omits references to city personnel (Connie, Sam) and abbreviations (amto, amg). The word cloud highlights water quality (algae, algal, bloom), infrastructure (canal, swale), and city service reply keywords (please, email, call).

In 75% of study weeks (31/41), the dominant algal taxon was cyanophyceae. Water temperatures seasonally peaked with temperatures in the high 20 °C to low 30 °C from May to October each year. Dissolved oxygen generally exhibited the opposite seasonal dynamics – with the highest and lowest levels, respectively, recorded in the winter (range: 6.9–10.0 mg/L) and summer (range: 2.94–6.86 mg/L). In contrast, the water’s pH was more basic in the winter and spring (range: 7.5–8.5) than summer and fall (range: 7.4–7.8). Specific conductance generally fluctuated between 347 and 594 umhos/cm, except for pronounced spikes in the winter of 2018–2019 and a large-isolated peak sampled during the week of May 13, 2019. USGS stream gauge data confirm that this spike reflects a large discharge event from upstream eutrophic Lake Okechobee, which generally increased downstream cyanobacteria and microcystin concentrations (Schaefer *et al*. 2020; USGS 2022).

Each 1 μg/L increase in waterborne microcystin-LR concentrations corresponded with 9% more algal 311 calls (95% confidence interval: 1.03–1.15, *p* = 0.002), after controlling for the number of algal 311 calls in the preceding week (Table 1). Higher water temperatures and/or more acidic conditions non-significantly increased algal distress calls.

## DISCUSSION

In this study, algal blooms and waterborne microcystin-LR were associated with residential algal complaints. Further categorization of the 311 call description fields revealed that a plurality of calls either reported or complained about the algal blooms. Notably, 5% of the calls explicitly discussed health symptoms (difficulty breathing, nausea, malaise), and an additional 7% mentioned noxious odors during prolonged exposure to cyanobacterial blooms. This suggests that inhalation of aerosolized cyanotoxins was the most likely exposure route since swimming or other water recreational activities were not explicitly reported by callers.

The present study’s results should be interpreted cautiously due to the relatively small sample size and uncertainty in exposure and 311 call complaints. Our retrospective study considered microcystin-LR levels from existing water quality surveillance. Only two published studies examined the correspondence between water and airborne microcystin levels or inhalation metrics. Most germane to our study, Schaefer *et al*. (2020) found a consistent relationship between human nasal and water microcystin levels in the neighboring St. Lucie River, FL – where water quality is also sensitive to Lake Okechobee’s discharge events (Schaefer *et al*. 2020). In contrast, Backer *et al*. (2010) found no relationship between cyanobacteria, microcystin, and aerosol concentrations (Backer *et al*. 2010).

Other processes may have also confounded the microcystin and 311 call association. During the study period (2018) and adjacent to the study area, a marine *K. brevis* bloom formed in the Gulf of Mexico (Metcalf *et al*. 2021). Red tide aerosolized brevetoxins were linked to self-reported respiratory symptoms and the toxins could be transported over distances greater than 4 km (Backer *et al*. 2003; Kirkpatrick *et al*. 2010). However, the present study found people residing in the zip codes adjacent to the Caloosahatchee River made nearly all 311 calls – suggesting this was the primary cause of algal complaints. Relatedly, the microcystin-LR and 311 relationship may reflect the inhalation of other compounds (e.g. trimethylamine from fish kills) that are associated with noxious odors over time.

Water quality monitoring and the 311 dispatch systems may be further integrated to improve public health surveillance. The 311 system could link to existing water quality dashboards that would alert callers if nearby HAB samples are at high levels (Florida Department of Environmental Protection 2020). Relatedly, the 311 dispatchers could create a standardized protocol/script to collect standardized information about potential HAB-related health symptoms. On the other hand, water quality sampling should prioritize collecting weekly water quality from a small number of stations throughout the calendar year. An automated water quality sampling system may help decrease labor costs (Corsi *et al*. 2014). Public health could longitudinally measure airborne toxic levels, biomarkers, or health symptoms from clusters of households living near water quality sampling sites. Future studies should consider cross-referencing 311 calls with poison control center calls which likely contain more reliable but potentially less common health outcome information (Figgatt *et al*. 2017). In summary, integrated environment and health surveillance systems can help focus resources to at-risk areas and proactively prepare health interventions.

Intuitively, the geographic distribution of 311 calls likely reflects both the phenomena of interest and differential 311 calling practices. Consistent with access to smartphone technology and experiences with the government, previously published studies suggest wealthier neighborhoods are more likely to call 311 across different municipalities (Clark *et al*. 2013). In a similar vein, neighborhoods with a higher proportion of minorities and low English proficiency speakers were less likely to call and/or report their residential address to 311 (Wang *et al*. 2017; Kontokosta & Hong 2021). Thus, 311 calls may report cyanobacterial blooms missed by existing water quality surveillance in higher socioeconomic positions and whiter neighborhoods. Cities should consider communication campaigns to increase 311 calls in neighborhoods with disproportionately low usage rates to increase constituent services.

## CONCLUSIONS

Monitoring HABs and potential adverse human health effects is notoriously difficult due to dynamic exposures. Non-emergency city service calls for government services may reflect cyanobacterial blooms-related health symptoms. Integrating the 311, environmental, and public health surveillance systems could provide callers with more information and target additional environmental and public health testing.

## Figures and Tables

**Table 1 |:** Statistical relationship between water quality conditions, temporal characteristics, and algal 311 calls during 2018–2019

Independent variable	OR	Std. Error	*p*-value	95% CI
Dissolved oxygen (mg/L)	1.18	0.20	0.32	0.84–1.66
Specific conductance (umhos/cm)	1.00	0.001	0.74	1.00–1.00
Temperature (°C)	1.11	0.07	0.08	0.98–1.26
pH, deviation from neutral	0.17	0.17	0.08	0.02–1.19
Microcystin-LR (μg/L)	1.09	0.03	0.002	1.03–1.15
Study year 2019 versus 2018	0.74	0.25	0.37	0.38–1.45
ln (preceeding week’s 311 algal call count +1)	2.02	0.26	<0.001	1.57–2.63

The results report the odds ratios (OR), standard errors (Std. Error), and 95% confidence intervals (CI).

## Data Availability

All relevant data are available from https://zenodo.org/record/4966946#.YiEAtJZOmuU.

## HIGHLIGHTS

- Integrating the 311, environmental, and public health surveillance systems could provide callers with more information and target additional testing.
- Cyanobacteria**’**s waterborne microcystin-LR concentrations were associated with the timing and number of algal complaints from local residents.
- People living in close proximity to prolonged and intense harmful algal blooms may experience respiratory health symptoms.
